# Association Between Abnormal Plasma Lipid Metabolism and Psychological Characteristics in Adolescents With Major Depressive Disorder

**DOI:** 10.1155/da/5564796

**Published:** 2025-05-12

**Authors:** Yuxin Wang, Hui Chen, Jinfeng Wang, Shurui Chen, Jiali Liu, Xianliang Chen, Sihong Li, Huajia Tang, Jiawei Zhou, Yanyue Ye, Yusheng Tian, Xiaoping Wang, Jiansong Zhou

**Affiliations:** ^1^Department of Psychiatry, The Second Xiangya Hospital of Central South University, Changsha, Hunan 410011, China; ^2^Bio-X Institutes, Shanghai Jiao Tong University, Shanghai, China

**Keywords:** childhood maltreatment, coping strategies, lipidomics, major depressive disorder, personality traits

## Abstract

**Background:** Major depressive disorder (MDD) in adolescents is a significant global public health issue, yet its pathophysiological mechanism remains unclear. Although changes in lipid metabolites have been observed in people with MDD, most relevant studies focus on adults, with limited research on adolescents. Furthermore, little is known about how interactions between metabolites and psychosocial factors contribute to MDD among adolescents. This study aimed to explore the relationship between lipid metabolism and psychological characteristics in adolescents with MDD to provide insights into its biological mechanisms.

**Methods:** A cross-sectional study was conducted on 40 adolescents with MDD patients and 20 healthy controls (HCs). Plasma samples were analyzed using ultra-performance liquid chromatography-tandem mass spectrometry (UPLC-MS/MS) for targeted lipidomics. Spearman analysis was employed to examine the correlation between differentially expressed lipids and the psychological characteristics of participants.

**Results:** We identified significant differences in 37 lipid species, including glycerophospholipids (GPs), glycerolipids (GLs), and sphingolipids (SPs), with a specific focus on lysophosphatidylcholine (LPC; 18:0), which demonstrated high diagnostic potential (area under the curve (AUC) = 0.886). Pathway analysis revealed significant disturbances in GP metabolism. Spearman correlation analysis showed that most differential lipid metabolites were negatively correlated with scores of depressive symptoms, childhood maltreatment (CM), extraversion, and neuroticism, while positively correlated with scores of psychoticism and positive coping in adolescents with MDD.

**Conclusions:** The study showed that adolescents with MDD presented a significantly differentiated profile of plasma lipidomics compared to HCs. These findings may contribute to a better understanding of the role of lipid metabolism in adolescent MDD.

## 1. Introduction

Major depressive disorder (MDD) is a significant public health issue. The World Health Organization (WHO) reports that 280 million people worldwide suffer from depression, including 23 million children and adolescents [1]. A global meta-analysis showed that the 1-year prevalence of adolescent MDD was 8%, with a lifetime prevalence of 19%. Additionally, 34% of adolescents aged 10–19 years are at risk of developing clinical depression [2]. Despite the significant rates, this condition is often misdiagnosed or overlooked in clinical settings and adolescents with MDD face limited treatment options and poor prognosis [2, 3]. Research indicates that the mechanisms of adolescent MDD differ from those of adult MDD [4, 5] and its underlying pathophysiology remains unclear.

Emerging evidence suggests that aberration of lipid metabolism and homeostasis may play a role in the pathophysiology of MDD [6–8]. Lipids are essential components of cell membranes and play significant roles in cellular signaling, energy storage, neural development, and brain function [9]. Studies show abnormalities in plasma lipid levels in MDD patients, including changes in cholesterol metabolism, phospholipid (PL) and sphingolipids (SPs) composition, and fatty acid profiles [10–12]. A meta-analysis revealed that, compared to healthy controls (HCs), MDD patients had higher triglyceride (TG) levels, but significantly lower levels of total cholesterol (TC) and very low-density lipoprotein (VLDL) [13]. Another study found that first-episode MDD patients had higher TG and lower high-density lipoprotein cholesterol (HDL-C) levels than HCs [14]. Ceramide (Cer) metabolism alteration have been observed, with species C18:0 and C20:0 most strongly linked to the disorder [15]. Targeted lipidomics also showed elevated plasma Cer levels in MDD patients, associated with more severe depressive symptoms [16]. However, most lipidomics investigations on MDD have focused on adults, even though adolescents exhibit distinct pathophysiological mechanisms and responses to antidepressants [17]. Existing studies have indicated disrupted polyunsaturated fatty acid (PUFA) metabolism in adolescents with MDD, with significantly lower omega-3 and omega-6 PUFAs than HCs [12, 18], suggesting unique lipid signatures in this group. However, few studies examine diverse lipid classes in adolescent MDD.

The biopsychosocial model posits that MDD result from a combination of biological, psychological, and social-environmental factors [19, 20]. Among these, childhood maltreatment (CM) has been confirmed as a key social-environmental risk factor for the development of adolescent MDD. Early meta-analysis has demonstrated that CM is linked to an earlier onset of depression, with affected individuals being 2.66–3.73 times more likely to develop this disorder [21]. CM may increase the risk of MDD in adulthood by disrupting the hypothalamic–pituitary–adrenal (HPA) axis and is linked to poorer illness prognosis and treatment outcomes [22, 23]. Perlman et al. [24] found that individuals with CM who later died by suicide exhibited significant dysregulation of fatty acids in the anterior cingulate cortex, specifically affecting the choline glycerophospholipid (GP) pool, which may lead to altered myelination and increased vulnerability to MDD. However, not all children and adolescents exposed to trauma develop depression. This highlights the importance of considering psychological factors, such as personality traits and coping strategies, in understanding the onset and progression of MDD. Neuroticism is a well-established risk factor for MDD, with high neuroticism linked to an increased risk of depression and comorbid anxiety disorders [25, 26]. Mendelian randomization studies suggest that neuroticism may play a causal role in the development of depression [27]. In contrast, extraversion appears protective, as individuals with higher extraversion tend to regulate emotions better and build stronger social support networks, reducing the risk of depression [28]. Research also indicates that extraversion may buffer the negative effects of neuroticism on depression [29]. Notably, Kolla et al. [30] found that higher trait neuroticism is associated with increased fatty acid amide hydrolase binding, suggesting a link between neuroticism and lipid metabolism in emotional regulation. Coping strategies, typically classified as positive or negative, also influence the outcome of depression. Negative coping strategies worsen depressive symptoms, while positive strategies help alleviate them [31]. Evidence indicates that CM may contribute to MDD through the mediating effects of personality traits and/or coping strategies [32, 33]. Children exposed to abuse are more likely to develop heightened neuroticism, which leads to negative coping strategies and increased vulnerability to MDD [34]. Despite evidence linking lipid metabolism to MDD, the interaction of lipid dysregulation, CM, personality traits, and coping strategies in adolescent MDD remains unclear.

Therefore, this study aims to: (1) identify plasma lipid metabolites that significantly differ between first-episode treatment-naïve adolescents with MDD and HCs, exploring potential diagnostic biomarkers for adolescent MDD; and (2) investigate the association between lipid metabolism, CM, personality traits, and coping strategies in adolescents with MDD, to identify significant factors affecting adolescent MDD and provide a reference for prevention and treatment.

## 2. Materials and Methods

### 2.1. Participants and Procedures

This cross-sectional study, part of the China Depression Cohort Study (CDCS), was conducted from January 1, 2019 to December 31, 2022. Adolescents with MDD were recruited from the outpatient psychiatry department of the Second Xiangya Hospital of Central South University using a continuous sampling method. All the participants were diagnosed by an attending psychiatrist or a senior specialist based on the Diagnostic and Statistical Manual of Mental Disorders, Fifth Edition (DSM-5). The inclusion criteria for adolescents with MDD were: (1) aged 14–24 years, (2) no history of psychiatric treatments (pharmacological, physical, or psychological), (3) MDD diagnosed based on DSM-5 criteria, and (4) normal intelligence with voluntary participation. HCs were recruited from schools and communities, matched to the MDD group for sex, age, and body mass index (BMI). The exclusion criteria for both groups included: (1) any other psychiatric disorders or comorbidities (e.g., neurodevelopmental disorders, schizophrenia, bipolar disorder, and substance-related and addictive disorders), (2) serious diseases affecting neurological, endocrine, hematological, cardiovascular, or other systems, and (3) any history of psychiatric treatments. After a thorough explanation of the study procedures, informed consent was obtained from all participants; for those aged under 18 years, parental consent was required. To confirm the diagnosis, all participants underwent a face-to-face interview with psychiatrists who had received standardized training, using the Structured Clinical Interview for DSM-5 (SCID-5). Participants then completed a sociodemographic questionnaire and a series of scales. Finally, 5 mL of fasting venous blood was collected from each participant.

### 2.2. Measurements

Demographic data, including sex, age, education level, and BMI were recorded. A 13-item version of the Beck Depression Inventory (BDI-13), a self-report scale, was used for the measurement of depressive symptoms over the past week [35]. The BDI-13 was chosen for its suitability in adolescents, as it is shorter than the 21-item version, thus, reducing participant fatigue while providing a reliable measure. Each item was measured on a four-point Likert scale (0 = not at all, 1 = mild, 2 = moderate, and 3 = severe), with higher total scores indicating more severe depressive symptoms. The Cronbach's alpha coefficient for the BDI-13 in this study was 0.89.

The Childhood Trauma Questionnaire (CTQ) was used to evaluate childhood trauma and neglect experiences [36]. This scale consists of 28 items that measure five types of CM: emotional abuse, emotional neglect, sexual abuse, physical abuse, and physical neglect. All the items are rated on a five-point Likert scale, ranging from 1 (never true) to 5 (very often true). Each of the five subscales contains five items, with scores ranging from 5 to 25 and the total CTQ score ranges from 25 to 125. The Cronbach's alpha of this scale was 0.80 in the present study.

Personality traits were evaluated using the Eysenck Personality Questionnaire (EPQ), based on Eysenck and Eysenck's [37] theory of personality. The EPQ measures four key dimensions: extraversion (E), neuroticism (N), psychoticism (P), and lie (L), with items answered “yes” or “no.” Each dimension has a specific set of questions and the subscale scores are summed separately.

The Simplified Coping Style Questionnaire (SCSQ) is a psychometric instrument used to assess coping strategies [38]. It consists of 20 items divided into two subscales: a positive coping subscale (12 items) and a negative coping subscale (eight items). All the items are rated on a four-point Likert scale from 0 (never) to 3 (very often), reflecting how frequently individuals use each coping strategy.

### 2.3. Sample Collection and Preparation

Five milliliters of fasting venous blood were drawn from the antecubital vein of all participants. Plasma samples were separated by centrifugation at 1500 g for 15 min and stored at −80°C for subsequent analysis. Targeted metabolomics profiling was performed by Metabo-Profile Corp. (Shanghai, China) [39]. After thawing in an ice bath, 10 μL of plasma/serum was added to a 96-well plate, followed by 300 μL of extraction solution in each well. The plate was vortexed for 20 min and then centrifuged at 4000 × *g* for 20 min; then, 20 μL of the supernatant was transferred to a new 96-well plate and mixed with 80 μL of diluting solution. The plate was sealed for liquid chromatography-mass spectrometry (LC-MS).

### 2.4. Lipidomics Analysis

Lipidomic analysis of plasma samples was performed using the Q300 Metabolite Array Kit obtained from Metabo-Profile Biotechnology (China), which enables absolute quantification of over 300 functional small molecule metabolites [39]. This approach allowed us to capture a broad spectrum of lipid species that are differentially regulated in MDD, rather than relying on predefined hypotheses about which lipids might be involved. The samples were analyzed using an ultra-performance LC-tandem MS (UPLC-MS/MS) system (ACQUITY UPLC-Xevo TQ-S, Waters Corp., Milford, MA, USA). The UPLC system was equipped with an ACQUITY UPLC BEH C18 1.7 µM VanGuard precolumn (2.1 mm × 5 mm) and an ACQUITY UPLC BEH C18 1.7 µM analytical column (2.1 mm × 100 mm), both maintained at 40°C. The sample manager temperature was set at 10°C. Mobile phases consisted of solvent A (ACN/water, 6:4, with 5 mM NH4FA and 0.1% FA) and solvent B (IPA/ACN, 9:1, with 5 mM NH4FA and 0.1% FA). Gradient elution conditions were as follows: 0–0.5 min (60% B), 0.5–3 min (60%–80% B), 3–7 min (80%–100% B), 7–9 min (100% B), 9–9.5 min (100%–60% B), and 9.5–11 min (60% B). The flow rate was set at 0.3 mL/min with an injection volume of 2 µL. The mass spectrometer was operated with a capillary voltage of 3 kV (ESI+), a source temperature of 150°C, a desolvation temperature of 550°C, and a desolvation gas flow rate of 1000 L/h. Quality control (QC) was performed by including test mixtures, internal standards, pooled biological samples, and conditioning and solvent blank samples to optimize instrument performance. Internal standards were added to the test samples to monitor analytical variations throughout the preparation and analysis process. The pooled QC samples, representing the biological average of the sample set, were injected at regular intervals (every 14 samples).

The raw data files generated by UPLC-MS/MS were processed using the TMBQ software (v1.0, Metabo-Profile, Shanghai, China) for peak integration, calibration, and quantitation of each metabolite. The preprocessed data underwent further univariate and multivariate statistical analyses. A two-sample Student's *t*-test or Wilcoxon rank-sum test was applied at univariate level, as appropriate. The Benjamini–Hochberg procedure was used for result correction, with a false discovery rate (FDR) set at 0.05. Further analysis was conducted using MetaboAnalyst software (https://www.metaboanalyst.ca), including principal component analysis (PCA), orthogonal partial least squares discriminant analysis (OPLS-DA), univariate analysis, and pathway analysis. PCA and OPLS-DA were conducted to visualize the alterations in lipid metabolism in adolescents with MDD and HCs after autoscaling. The variable importance in the projection (VIP) value of each variable was calculated in the OPLS-DA model to assess its contribution to classification. Lipids with VIP > 1.0 and FDR < 0.05 were considered significant differential lipids.

### 2.5. Statistical Analysis

Numerical variables were presented as mean ± standard deviation (SD), while categorical variables were expressed as quantities and percentages (%). The normality of the numerical data was tested using the Shapiro–Wilk test. Since most of the data did not meet the normality assumption, the Mann–Whitney *U* test was used to compare the age, years of education, BMI, and scale scores between patients with MDD and HCs. The chi-square (*χ*^2^) test was used to compare the gender distribution between the two groups. BMI was calculated as weight divided by height squared (kg/m^2^).

Receiver operating characteristic (ROC) curve analysis was conducted to assess the diagnostic performance of individual lipids. A forward stepwise binary logistic regression analysis was performed for multivariate analyses. Spearman correlation analysis was employed to examine the correlation between differentially expressed lipids and psychological variables in the MDD group. Factors potentially influencing metabolism, such as gender, age, and BMI, were used as covariates. A *p*-value lower than 0.05 (two-tailed) indicated statistical significance. All statistical analyses were conducted using IBM SPSS Statistics software version 25.0 for Windows, Graphpad Prism 9.1.0, and MetaboAnalyst 6.0.

## 3. Result

### 3.1. Characteristics of Participants

The sociodemographic and psychological characteristics of the 40 patients with MDD (25 females and 15 males) and 20 HCs (12 females and 8 males) are summarized in Table 1. There were no significant differences between the two groups in terms of age, gender, level of education, and BMI (*p* > 0.05). In the MDD group, the mean depression score (BDI-13) was 18.7 ± 7.2. Regarding childhood trauma (CTQ), the total score was 53.8 ± 12.8, with the emotional abuse having the highest scores (11.6 ± 4.7) among all subscales. In terms of personality traits (measured by EPQ), the mean score of extraversion was 6.3 ± 3.7, neuroticism was 5.3 ± 4.6, and psychoticism was 7.1 ± 3.2. For coping strategies (SCSQ), the score was 15.7 ± 6.4 for positive coping and 12.9 ± 3.8 for negative coping.

### 3.2. Lipidomics Analysis

The targeted lipidomics analysis of plasma samples from 40 patients with MDD and 20 HCs detected a total of 272 lipid molecules. Among these, 62 were glycerolipids (GLs), including 53 triacylglycerols (TAGs) and nine diacylglycerols (DAGs). Forty lipids belonged to SPs, comprising 28 sphingomyelins (SMs), 11 Cers, and one Cer phosphoethanolamine (CerPE). The remaining lipids were GPs, covering 73 phosphatidylcholines (PCs), 28 phosphatidylethanolamines (PEs), 13 phosphatidylinositols (PIs), 13 phosphatidylserines (PSs), one phosphatidic acid (PA), 17 lysophosphatidylcholines (LPCs), and eight lysophosphatidylethanolamines (LPEs).

**Figure 1:**
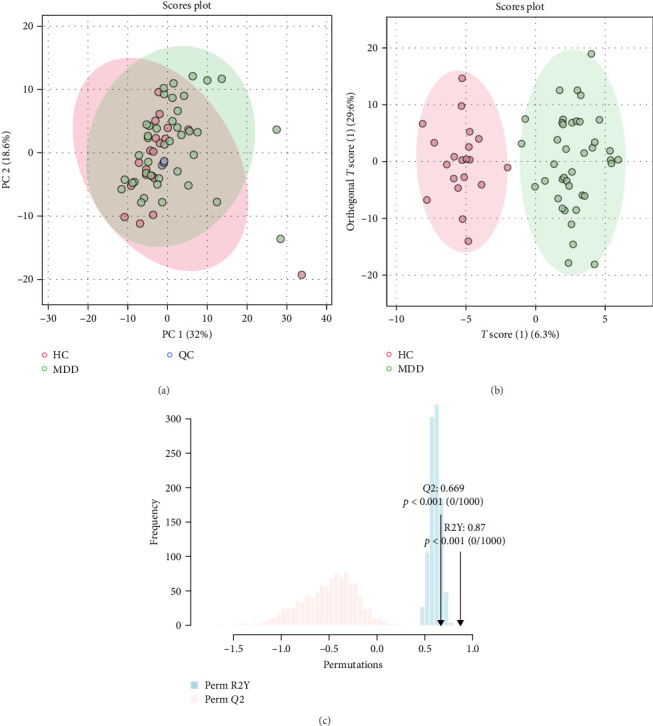
Principal component analysis (PCA) and orthogonal partial least squares discriminant analysis (OPLS-DA) results. (A) PCA score plot for the MDD and HC groups. (B) OPLS-DA score plot comparing the MDD and HC groups. (C) Permutation testing results of the OPLS-DA model, showing the distribution of *R*^2^ and *Q*^2^ values with their corresponding *p*-values.

The detected lipids were analyzed using MetaboAnalyst 6.0. After autoscaling, the QC samples clustered tightly in the PCA score plot (Figure 1A), indicating stable instrument performance and reliable data quality. The OPLS-DA model revealed significant separation between the MDD and HC groups after log transformation and Pareto scaling (Figure 1B). The 1000 permutation test yielded *Q*^2^ = 0.669 with a *p*-value < 0.001 (Figure 1C), indicating that the model was not overfitting.

**Figure 2:**
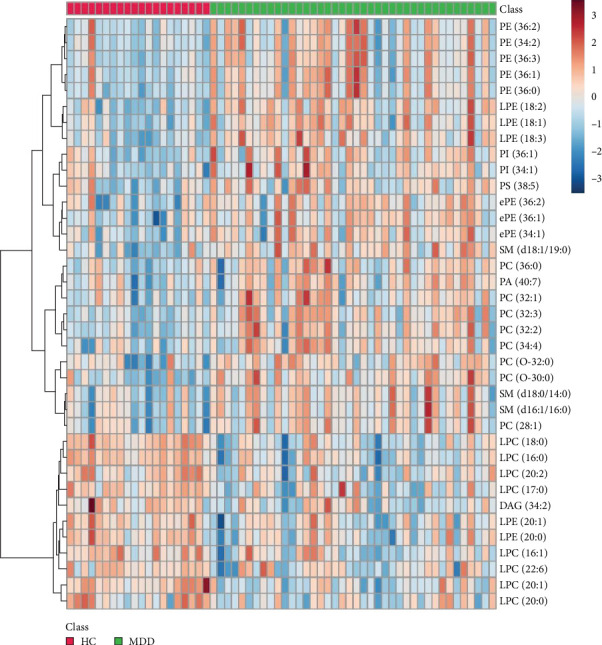
Heatmap of the differential lipid metabolites in adolescents with MDD vs. HCs. HC, healthy controls; MDD, major depressive disorder.

The lipids differentiated between the two groups were selected using the criteria of FDR < 0.05 and VIP > 1, which identified a total of 37 significant differential lipids, with 11 downregulated and 26 upregulated in the MDD group. Detailed information on these lipids is provided in Table 2. The heatmap of differential lipids between the MDD and HC groups is shown in Figure 2.

### 3.3. Pathway Analysis

**Figure 3:**
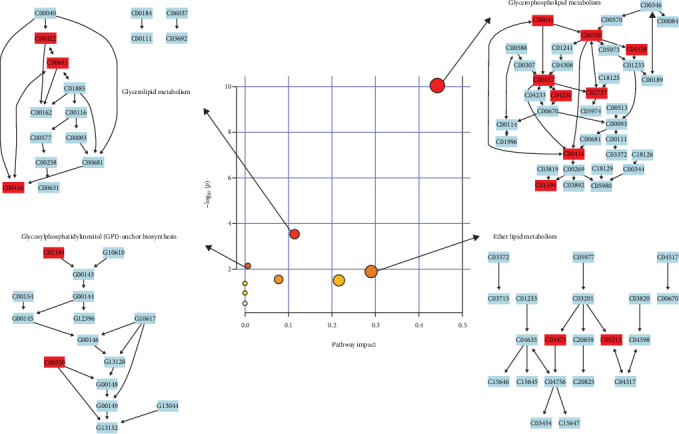
Bubble plot and pathway diagram of pathway analysis.

Pathway analysis of lipid metabolites was performed using MetaboAnalyst 6.0 to further examine the metabolic disturbances in the MDD and HC groups. Following data normalization and identification of key metabolites, the metabolites were mapped to metabolic pathways using the Human Metabolome Database (HMDB) and KEGG IDs. Enrichment analysis, including hypergeometric tests and pathway impact scores, identified significantly affected pathways. The results were visualized using pathway maps and bubble plots, highlighting metabolic alterations linked to depression. The significant pathways are presented in Table 3 and Figure 3.

Significantly altered pathways included GP metabolism, GL metabolism, glycosylphosphatidylinositol (GPI)-anchor biosynthesis, and ether lipid metabolism (Table 3). The extent of disturbances in these pathways is shown in Figure 3, with the size and color of the bubbles indicating the impact and significance of pathways. GP metabolism showed the highest impact, followed by GL metabolism, GPI-anchor biosynthesis, and ether lipid metabolism, all indicating significant alterations in lipid metabolism in the MDD group.

### 3.4. Biomarkers Related to Adolescent MDD

**Figure 4:**
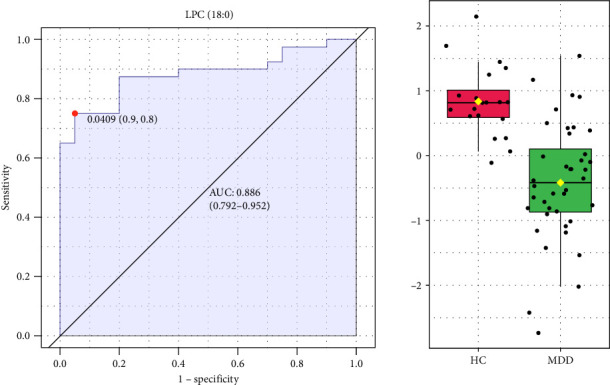
ROC curves for LPC (18:0). AUC, area under the curve; LPC (18:0), lysophosphatidylcholine (18:0); ROC, receiver operating characteristic.

To identify potential diagnostic biomarkers for adolescent MDD, we performed ROC analysis and forward stepwise binary logistic regression analysis to assess the diagnostic value of 37 significant differential lipids. The ROC analysis showed that LPC (18:0) had a high diagnostic value for distinguishing patients with MDD from HCs, with the largest area under the curve (AUC) of 0.886 among all lipids (Figure 4). Logistic regression showed that LPC (18:0) was a significant predictive factor for MDD (*p* < 0.001) and that decreased plasma LPC (18:0) was associated with an increased risk of MDD (Exp (*β*) = 0.753, 95% CI: 0.649–0.873; Table 4).

### 3.5. Correlation Analysis

**Figure 5:**
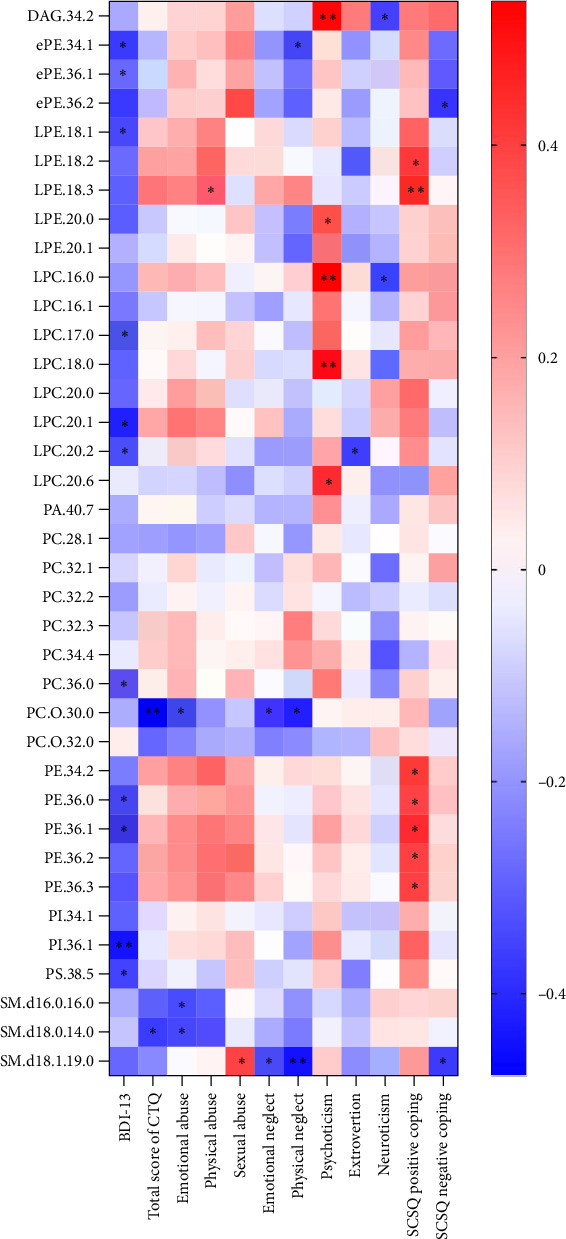
Heatmap of Spearman correlation between differential lipid metabolites and psychological scales scores. BDI, Beck Depression Inventory; CTQ, Childhood Trauma Questionnaire; SCSQ, Simplified Coping Style Questionnaire. *⁣*^*∗*^*p* < 0.05; *⁣*^*∗∗*^*p* < 0.01.

Spearman correlation analysis was performed to explore the association of differential lipid metabolites with scores of BDI-13, CTQ, EPQ, and SCSQ in the MDD group. We found that most lipid metabolites were negatively correlated with scores of BDI-13, CTQ, extraversion, and neuroticism, while positively correlated with scores of psychoticism and positive coping strategies (Figure 5). Specifically, the levels of 11 lipid metabolites, including PI (36:1), LPC (20:1), and PE (36:1), were significantly negatively correlated with the BDI-13 score. In addition, PC (O-30:0) and SM lipids were negatively associated with the total CTQ score and scores of certain subscales (emotional/physical neglect and abuse). Psychoticism was positively correlated with lipids such as LPC (18:0) and DAG (34:2), whereas neuroticism showed a negative association with DAG (34:2). LPC (20:2) was exclusively negatively associated with extroversion. Furthermore, PE lipids were primarily positively associated with the score of positive coping strategies in SCSQ.

## 4. Discussion

This study explored the lipidomic profiles of adolescents diagnosed with MDD through targeted lipidomics analysis using UPLC-TQ/MS and identified significant differential lipid metabolites that could serve as potential biomarkers for MDD. The findings revealed that specific lipid species, particularly GLs, GPs, and SPs, were differentially express between patients with MDD and HCs, with LPC (18:0) emerging as a potentially effective and specific biomarker for diagnosing MDD in adolescents. Furthermore, pathway analysis showed that these differentially expressed lipids were mainly involved in GP metabolism and correlation analysis revealed the complex associations between lipid metabolites and psychological measures such as childhood trauma, personality traits, and coping strategies.

Based on the lipidomics analysis, adolescents with MDD exhibited distinct lipid profiles compared to HCs, including lower levels of DAGs (e.g., DAG (34:2)) and LPCs (particularly LPC (17:0) and LPC (18:0)) as well as higher levels of PC, PE, and unsaturated SM. These findings indicate disruptions in GL, GP, and SP metabolism pathways, which are crucial for maintaining cell membrane integrity, signaling, and neuroinflammatory balance. Previous studies on metabolomics in adolescent MDD have focused on different aspects compared to our research. A recent study by Gan et al. [40] found that amino acid and lipid metabolism are significantly disrupted in adolescents with MDD. Notably, alterations in the metabolism of GP and tyrosine may serve as potential biomarkers differentiating between first-episode drug-naïve MDD and treatment-resistant depression [40]. Wang et al. [41] observed increased levels of cholesterol, SM, and Cer and decreased levels of ether lipids in adolescents with MDD. Interestingly, both our results and existing studies on adolescent MDD differed from previous findings in adults with MDD. A pioneering study by Demirkan et al. [42] used unsupervised lipidomic analysis of plasma lipids in 742 individuals, finding significant negative correlations between depressive symptoms scores and SM 23:1/SM 16:0 ratio and the molar proportion of PC O 36:4 and its ratio to Cer 20:0. Absolute levels of PC O 36:4 were also negatively correlated with depressive symptoms in an independent validation with 753 individuals [42]. Liu et al. [43] used UPLC-Q-TOF/MS to analyze plasma lipids in MDD patients, finding decreased levels of acyl carnitines, ether lipids, and tryptophan, and increased LPCs, LPEs, and PEs. In their subsequent study, they observed significant increases in LPC, LPE, PC, PE, PI, and TG in MDD, and decreases in 1-O-alkyl-2-acyl-PE (PE O) and SM with odd summed carbon number [44]. These discrepancies might be attributed to the influence of age on lipid metabolism. Our study focused on adolescents aged 14–24 years, in line with the work of Sawyer et al. [45], which suggests that the definition of adolescence should extend to 24 years due to ongoing biological growth and delayed social transitions. Adolescents are in a period of growth and development, with significant hormonal fluctuations and more active lipid metabolism and their metabolic pathways are not yet fully stable, leading to lipidomic characteristics that differ from those of adults.

The most significant pathway identified was GP metabolism, which plays a critical role in cell membrane structure, signaling, and energy balance. Evidence from animal studies has demonstrated that disruptions in GP metabolism are strongly associated with depressive-like behaviors. A comprehensive multiomics analysis revealed significant alterations in GP metabolism in depressed mice, with a notable increase in differential lipid metabolites linked to gut microbiota changes [46]. Another study found that altered fecal metabolites and colonic GPs were associated with gut microbiota imbalance in a mouse model of depression, suggesting that gut microbiota changes might contribute to MDD by modulating GP metabolism [47]. Furthermore, Xu et al. [48] found that oral D-ribose might induce depressive-like behaviors in mice by disrupting the intestinal epithelial barrier and altering GP metabolism, with genera such as Lachnospiraceae closely associated with changes in GP profiles. Collectively, these animal studies suggest that GP metabolism may be a key mediator between gut-brain axis dysfunction and MDD, with microbial imbalance potentially triggering depressive symptoms through its impact on GP metabolism. In clinical studies, disturbances in GP metabolism have also been reported in patients with MDD. For instance, Yu et al. [49] found that GP alterations might contribute to the onset of depressive and anxiety-like behaviors. Similarly, Liu et al. [43] observed significant alterations in plasma GP metabolites in MDD patients, linking them to symptom severity. They proposed that disturbances in GP metabolism could affect cellular membrane dynamics and signaling pathways, which might contribute to the pathophysiology of MDD [43]. Overall, these findings highlight the central role of GP metabolism in MDD and its broad impact on depression-related mechanisms.

Many previous studies employed lipidomics techniques to identify specific biomarkers for MDD. Liu et al. [44] identified a combinational lipid panel (LPE 20:4, PC 34:1, PI 40:4, SM 39:1, 2, and TG 44:2) as potential biomarker with an AUC range of 0.855–0.931, demonstrating good sensitivity and specificity. Kim et al. [50] extracted serum lipid panels that differentiated between pairs of groups: LPA (16:1), TG (44:0), and TG (54:8) distinguished current MDD from HCs with 76% accuracy; LPC (16:1), TG (44:0), TG (46:0), and TG (50:1) distinguished current MDD from remitted MDD at 65% accuracy; and LPA (16:1), TG (52:6), TG (54:8), and TG (58 : 10) distinguished remitted MDD from HCs with 60% accuracy. Wu et al. [51] identified cholesterol sulfate (AUC: 0.823) and PC (18:2 (2E, 4E)/0:0; AUC 0.778) as potential biomarkers for antenatal depression in women who underwent cesarean section. However, research on biomarkers for adolescent MDD is limited. In our study, we identified LPC (18:0) as a potential biomarker for the diagnosing MDD in adolescents. The high AUC value and significant regression results suggests LPC (18:0) could not only serves as a potential biomarker for early detection but also a therapeutic target for restoring lipid homeostasis in patients with MDD. Our previous metabolomics study identified pyruvate, malic acid, and linoleylcarnitine as potential biomarkers for MDD [12]. Lipid biomarkers, together with polar metabolite biomarkers, could enhance the accuracy of clinical diagnosis for MDD.

LPCs are integral to cellular function, widely present on cell membranes where they influence membrane permeability, fluidity, and protein function [52]. LPCs modulate various cellular signaling pathways. When LPC binds to its receptors, such as G protein-coupled receptor 4, it activates downstream cascades, including the protein kinase C (PKC) pathway, influencing neuronal activity and synaptic plasticity. LPC can also be converted to LPA by lysophosphatidic acid acyltransferase (LPAAT), which transfers a fatty acid residue from the sn-2 to the sn-1 position. LPA is a bioactive lipid that activates multiple signaling pathways through its receptors (e.g., LPA1, LPA2, LPA3, and LPA4) [45]. Reduced LPC levels may impair neuronal membrane integrity and neurotransmitter signaling, contributing to cognitive impairment in MDD patients [50]. In addition, LPC acts as a pro-inflammatory mediator, activating immune cells and stimulating cytokine release (e.g., interleukin-6 (IL-6) and TNF-*α*) [53], which can disrupt the blood–brain barrier (BBB) and promote central nervous system inflammation [54]. LPCs also regulate mood-related neurotransmitters, such as serotonin and dopamine. Moreover, LPCs play a key role in the function of the BBB, helping transport PUFAs, especially docosahexaenoic acid (DHA), into the brain [55, 56]. DHA, an omega-3 fatty acid, has antidepressive and antianxiety effects by modulating neurotransmitter metabolism and reducing neuroinflammation [57]. MDD patients often have lower DHA levels and DHA supplementation may alleviate depressive symptoms [58]. LPC-bound DHA transport across the BBB is facilitated by MFSD2a [55, 56]; reduced LPC levels can impair this process, leading to lower DHA levels in the brain, neuronal cell loss, and cognitive deficits.

To better understand the relationship between differential lipids and depression severity, a correlation analysis was conducted between identified differential lipids and the score of BDI-13. Multiple lipids showed significant negative correlations with the BDI-13 score, indicating that lipid metabolism disorders in MDD were closely related to the severity of depression. Lipids such as LPC and PE are essential for maintaining cell membrane structure and supporting neurotransmitter signaling, suggesting their potential protective role in mood regulation and neural function [50]. Reduced levels of these lipids may impair neuroplasticity and synaptic function, leading to cognitive and emotional dysregulation commonly observed in MDD patients. Similarly, the negative associations of PC (O-30:0) and SM lipids with CTQ scores, particularly concerning emotional and physical neglect or abuse, suggest that early-life trauma may have long-lasting effects on lipid metabolism. Previous studies suggest that dysregulated lipid metabolism precedes the onset of psychosis [59, 60], implying that CM may contribute to the development of depression by disrupting lipid metabolism.

Further exploration of the association between differential lipids and personality traits revealed negative correlations between neuroticism and several lipids, such as DAG (34:2) and LPC (16:0). Studies have found that high neuroticism is associated with elevated levels of IL-6 [61] and a significant increase in pro-inflammatory cytokines has also been observed in patients with MDD who experienced CM [62, 63]. This suggests that high neuroticism and the experience of CM may contribute to MDD through neuroinflammatory mechanisms, potentially leading to the degradation of protective lipids and the onset of the disorder. Notably, the positive associations between PE lipids and positive coping strategies, as measured by the SCSQ, further support the notion that effective coping mechanisms are associated with healthier lipid profiles. PE plays a crucial role in membrane dynamics and brain health, and individuals employing adaptive coping strategies may be better equipped to regulate stress responses, thereby minimizing neuroinflammation and maintaining lipid balance [64]. This could buffer the negative impact of stress and emotional dysregulation on metabolic processes, providing protection effect against worsening depressive symptoms.

Our study has several limitations. First, the sample size is small, which may limit the generalizability of the results. Future studies with larger sample sizes and more representative and diverse samples are required to improve the reliability of the findings. Furthermore, the cross-sectional design prevents us from establishing a causal relationship between lipid alterations and MDD. Longitudinal studies are needed to determine whether these lipidomic changes contribute to the onset of MDD and to explore their potential as predictive biomarkers.

## 5. Conclusion

This study found significant differences in plasma lipid metabolites between adolescents with MDD and HCs, identifying LPC (18:0) as a potential biomarker for adolescent MDD. The study also highlights that significant differential lipids are strongly associated with scores of BDI-13, CM, EPQ, and SCSQ, providing insights into how biopsychosocial factors relate to MDD. Overall, this study may improve our understanding of the pathogenesis of adolescent MDD. Future longitudinal studies with larger, more representative, and diverse samples are needed to confirm the current findings and explore the mechanisms involved.

## Figures and Tables

**Table 1:** Clinical characteristics of adolescents with MDD and HCs.

Characteristics	MDD (*n* = 40)	HC (*n* = 20)	*χ* ^2^/U	*p*-Value
Sociodemographic characteristics				
Gender, *n* (%)	—	—	0.04	0.85
Male	15 (37.5%)	8 (40%)	—	—
Female	25 (62.5%)	12 (60%)	—	—
Age	18.4 ± 2.8	18.0 ± 0.7	−0.16	0.87
Level of education	12.3 ± 2.7	12.0 ± 1.0	−2.65	0.62
BMI	19.9 ± 2.7	21.2 ± 3.8	−1.17	0.24
Psychological characteristics				
Depression symptoms (BDI-13)	18.7 ± 7.2	NA	NA	—
Childhood trauma (CTQ)	53.8 ± 12.8	NA	NA	—
Emotional abuse	11.6 ± 4.7	NA	NA	—
Physical abuse	7.2 ± 3.3	NA	NA	—
Sexual abuse	5.7 ± 1.3	NA	NA	—
Emotional neglect	18.7 ± 4.4	NA	NA	—
Physical neglect	10.7 ± 3.2	NA	NA	—
Personality traits (EPQ)				
Extraversion (E)	6.3 ± 3.7	NA	NA	—
Neuroticism (N)	5.3 ± 4.6	NA	NA	—
Psychoticism (P)	7.1 ± 3.2	NA	NA	—
Coping strategies (SCSQ)				
Positive coping	15.7 ± 6.4	NA	NA	—
Negative coping	12.9 ± 3.8	NA	NA	—

*Note:* Continuous variables were expressed as mean ± SD and qualitative variables were expressed as *n* (%). NA indicates data is not available for the healthy control group.

Abbreviations: BDI, Beck Depression Inventory; BMI, body mass index; CTQ, Childhood Trauma Questionnaire; EPQ, Eysenck Personality Questionnaire; HCs, health controls; MDD, major depressive disorder; SCSQ, Simplified Coping Style Questionnaire; SD, standard deviation.

**Table 2:** Significant differential lipids between the MDD and HC groups.

Lipid	Class	FC	FDR	VIP	Pathway
DAG (34:2)	DAG	0.61	2.25E-02	1.38	Glycerolipid metabolism
LPC (16:0)	LPC	0.85	5.26E-05	2.05	Glycerophospholipid metabolism
LPC (16:1)	LPC	0.81	6.85E-03	1.51	Glycerophospholipid metabolism
LPC (17:0)	LPC	0.75	4.47E-03	1.30	Glycerophospholipid metabolism
LPC (18:0)	LPC	0.72	2.07E-05	2.40	Glycerophospholipid metabolism
LPC (20:0)	LPC	0.84	1.72E-02	1.75	Glycerophospholipid metabolism
LPC (20:1)	LPC	0.74	7.85E-04	2.05	Glycerophospholipid metabolism
LPC (20:2)	LPC	0.76	4.43E-03	1.96	Glycerophospholipid metabolism
LPC (22:6)	LPC	0.74	4.64E-02	1.38	Glycerophospholipid metabolism
LPE (18:1)	LPE	1.75	1.16E-03	1.96	Glycerophospholipid metabolism
LPE (18:2)	LPE	1.88	5.53E-04	1.94	Glycerophospholipid metabolism
LPE (18:3)	LPE	1.96	7.85E-04	1.92	Glycerophospholipid metabolism
LPE (20:0)	LPE	0.66	1.12E-02	1.72	Glycerophospholipid metabolism
LPE (20:1)	LPE	0.72	2.35E-02	1.60	Glycerophospholipid metabolism
PA (40:7)	PA	1.53	3.07E-02	1.31	Glycerophospholipid metabolism
PC (28:1)	PC	1.15	4.52E-02	1.83	Glycerophospholipid metabolism
PC (32:1)	PC	1.43	3.58E-02	1.24	Glycerophospholipid metabolism
PC (32:2)	PC	1.59	7.01E-04	1.96	Glycerophospholipid metabolism
PC (32:3)	PC	1.87	5.97E-03	1.89	Glycerophospholipid metabolism
PC (34:4)	PC	1.61	3.23E-02	1.53	Glycerophospholipid metabolism
PC (36:0)	PC	1.40	2.73E-02	1.26	Glycerophospholipid metabolism
PE (34:2)	PE	1.51	4.35E-02	1.56	Glycerophospholipid metabolism
PE (36:0)	PE	1.68	2.03E-03	1.50	Glycerophospholipid metabolism
PE (36:1)	PE	1.63	1.14E-02	1.37	Glycerophospholipid metabolism
PE (36:2)	PE	1.53	1.70E-02	1.36	Glycerophospholipid metabolism
PE (36:3)	PE	1.88	2.35E-03	1.82	Glycerophospholipid metabolism
PI (34:1)	PI	1.38	4.75E-02	1.05	Glycerophospholipid metabolism
PI (36:1)	PI	1.67	1.27E-02	1.49	Glycerophospholipid metabolism
PS (38:5)	PS	1.25	3.78E-02	1.23	Glycerophospholipid metabolism
SM (d16:1/16:0)	SM	1.22	3.72E-02	1.77	Sphingolipid metabolism
SM (d18:0/14:0)	SM	1.25	3.35E-02	1.74	Sphingolipid metabolism
SM (d18:1/19:0)	SM	1.51	1.00E-03	1.83	Sphingolipid metabolism
PC (O-30:0)	PC	1.53	1.09E-03	2.10	Other
PC (O-32:0)	PC	1.21	1.27E-02	1.60	Other
ePE (34:1)	PE	1.32	1.94E-02	1.64	Other
ePE (36:1)	PE	1.50	1.27E-02	1.71	Other
ePE (36:2)	PE	1.49	3.84E-02	1.26	Other

Abbreviations: DAG, diacylglycerol; FC, fold change; FDR, false discovery rate; LPC, lysophosphatidylcholine; LPE, lysophosphatidylethanolamine; PA, phosphatidic acid; PC, phosphatidylcholine; PE, phosphatidylethanolamine; PI, phosphatidylinositol; PS, phosphatidylserine; SM, sphingomyelin; VIP, variable importance in the projection.

**Table 3:** Significantly altered pathways in adolescents with MDD.

Pathway	Total	Hits	Raw P	Holm P	FDR	Impact
Glycerophospholipid metabolism	36	8	8.95E−11	7.16E-09	7.16E−09	4.42E−01
Glycerolipid metabolism	16	3	2.93E−04	2.31E-02	1.17E−02	1.13E−01
Glycosylphosphatidylinositol (GPI)-anchor biosynthesis	15	2	7.21E−03	5.63E-01	1.92E−01	6.39E−03
Ether lipid metabolism	20	2	1.27E−02	9.80E-01	2.55E−01	2.89E−01

*Note:* Hits, number of significantly altered lipids in the pathway; Holm *p*-value, *p*-value corrected using the Holm–Bonferroni method; Impact, pathway impact score; Raw *p*-value, raw *p*-value before correction; Total, total number of lipids in the pathway.

Abbreviation: FDR, false discovery rate.

**Table 4:** Logistic regression analysis of lipid biomarkers in adolescent with MDD.

Step	Variable	*B*	Standard error	*p*-Value	Exp (*B*)	95% confidence interval
Step 1^a^	LPC (18:0)	−0.284	0.076	0.001	0.753	0.649–0.873
	Constant	8.997	2.311	0.001	8078.001	NA

Step 2^b^	LPC (18:0)	−0.670	0.218	0.002	0.512	0.334–0.784
	PC (36:0)	1.999	0.684	0.003	7.384	1.932–28.221
	Constant	8.318	3.696	0.024	4096.474	NA

Step 3^c^	LPE (18:1)	187.635	13653.530	0.989	3.081E + 81	NA
	LPC (18:0)	−10.477	768.797	0.989	0.001	NA
	PC (36:0)	26.418	2007.504	0.990	2.97E + 11	NA
	Constant	52.250	12394.740	0.997	4.92E + 22	NA

^a^The variable included in Step 1: LPC (18:0).

^b^The variable included in Step 2: PC (36:0).

^c^The variable included in Step 3: LPE (18:1).

## Data Availability

The data that support the findings of this study are available from the corresponding author upon reasonable request.
